# Current-controlled periodic double-polarity reversals in a spin-torque vortex oscillator

**DOI:** 10.1038/s41598-024-74094-0

**Published:** 2024-10-15

**Authors:** Chloé Chopin, Simon de Wergifosse, Anatole Moureaux, Flavio Abreu Araujo

**Affiliations:** Institute of Condensed Matter and Nanosciences, Université catholique de Louvain, Place Croix du Sud 1, 1348 Louvain-la-Neuve, Belgium

**Keywords:** Applied physics, Magnetic devices

## Abstract

Micromagnetic simulations are used to study a spin-torque vortex oscillator excited by an out-of-plane dc current. The vortex core gyration amplitude is confined between two orbits due to periodical vortex core polarity reversals. The upper limit corresponds to the orbit where the vortex core reaches its critical velocity triggering the first polarity reversal which is immediately followed by a second one. After this double polarity reversal, the vortex core is on a smaller orbit that defines the lower limit of the vortex core gyration amplitude. This double reversal process is a periodic phenomenon and its frequency, as well as the upper and lower limit of the vortex core gyration, is controlled by the input current density while the vortex chirality determines the apparition of this confinement regime. In this non-linear regime, the vortex core never reaches a stable orbit and thus, it can be of interest for neuromorphic application as a leaky integrate-and-fire neuron for example.

## Introduction

**Fig. 1:**
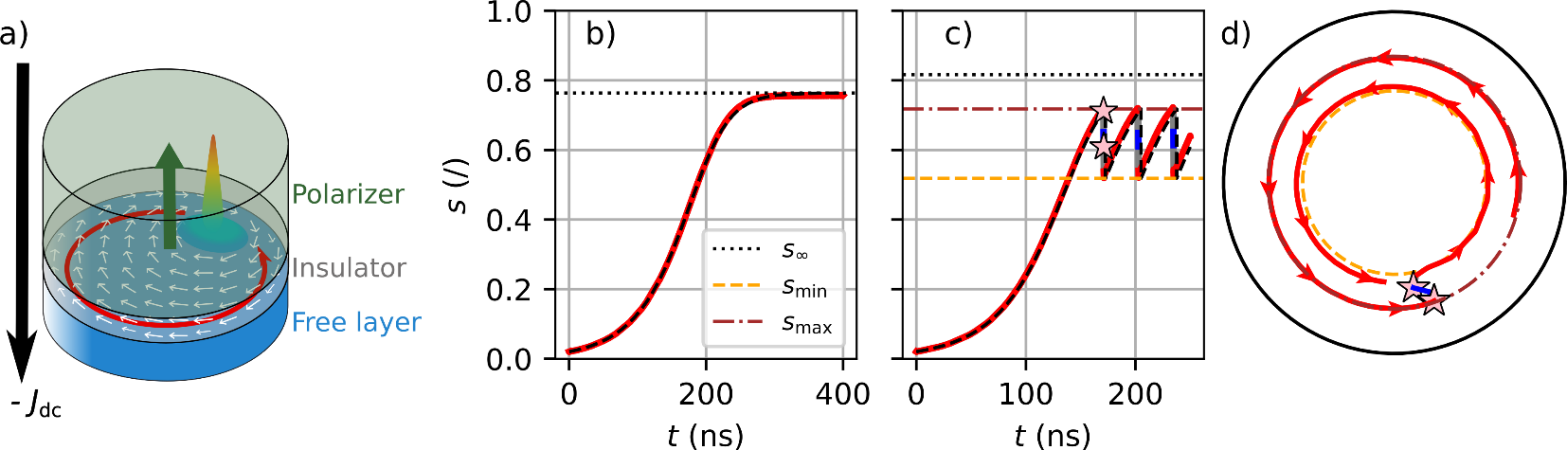
(**a**) Schematic diagram of an STVO with the polarizer in green with a green arrow repesenting its magnetization direction. The insulator is in gray and the free layer with a magnetic vortex as a magnetic ground state is in blue. The vortex has a positive polarity ($$P=+$$1) and a negative chirality ($$C=-$$1). In-plane magnetization is represented by white arrows whereas the red arrow represents the gyrotropic motion of the vortex core. (**b**) Evolution of the reduced vortex core position $$s(t)$$ in the steady-state regime saturating at $$s_\infty$$ with $$J_\text {dc}$$$$=-$$5.0 MA/cm^2^. The dashed line is the prediction using Eq. (1). (**c**) Evolution of $$s(t)$$ in the confinement regime with $$J_\text {dc}$$$$=-$$6.0 MA/cm^2^. The trajectory is plotted in red (resp. blue) for a positive (resp. negative) polarity. The black dashed line is the estimation of $$s(t)$$ using Eq. (3). Pink stars indicate polarity reversals. (**d**) Trajectory of the vortex core inside the magnetic dot while in the confinement regime. The vortex core motion direction is represented by arrows. When the polarity is negative, the gyration sense is reversed.

A magnetic vortex is a topological structure with a curling in-plane magnetization except at the vortex core where the magnetization points out-of-plane (see Fig. 1a). Its polarity *P* is positive ($P = +$1) when the out-of-plane (OOP) magnetization is pointing up and negative otherwise ($P = -$1). Its chirality *C* defines the curling in-plane magnetization orientation which is either clockwise ($C=-$1) or counterclockwise ($C=+$1). These two parameters impact the vortex core gyrotropic motion as its polarity determines the sense of the vortex core gyration as well as the apparition of sustained oscillations while there is a splitting of its dynamics depending on its chirality^1,2^. The vortex polarity can be reversed by applying different magnetic fields like static^3,4^, pulses^5–8^ or oscillating^9–11^ OOP magnetic fields as well as oscillating^12^ or rotating^13^ in-plane magnetic fields. The vortex polarity can also be reversed by a variety of spin-polarized currents like in-plane ac currents^14,15^ or pulses^16^ as well as out-of plane dc current^17^. Depending on the excitation, multiple polarity reversals can occur^8,14,16^ as in a nanocontact where a periodic polarity reversal is obtained with an input dc current^18,19^. The reversals occur in self-sustained oscillations leading to chaotic oscillations that may be interesting for secure communications^18^.

Here, a spin-torque vortex oscillator (STVO) is studied (see Fig. 1a). The device is composed of two magnetic layers decoupled by a non-magnetic insulator. The first magnetic layer, the polarizer, has a fixed magnetization, while the second layer, the free layer, has a vortex as its ground state due to the magnetic dot’s geometry^20^. An input current density is injected in the STVO to trigger gyrotropic motion of the vortex core. Indeed, the input current is spin-polarized by the polarizer and applies a spin-transfer torque on the free layer leading to sustained oscillations if the following conditions are met: $J_\text {dc} P p_z <$ 0 and $|J_\text {dc} | \ge |J_\text {c1} |$ with $J_\text {dc}$ the input current density, *P* the vortex polarization, $p_z$ the OOP magnetization of the polarizer and $J_\text {c1}$ the critical current for sustained oscillations. In a nanocontact, the Ampère-Oersted field (AOF) generated by the input current favours a magnetic vortex, thus, the vortex chirality tends to align parallel to the AOF. However, in an STVO, its initial chirality can be set deterministically^21^, thus its influence on the periodic reversal process can be studied.

Two distinct regimes^2,22^ arise depending on the vortex chirality and polarity, the polarizer orientation and the input current density: the resonant regime where the vortex core damps back to the magnetic dot center and the auto-oscillating regime where the vortex core reaches a stable orbit as in Fig. 1b. Here, a third regime^18^ is observed as shown in Fig. 1c and d. Indeed, when the vortex core reaches the upper orbit $s_\text {max}$, its polarity is reversed. This first polarity reversal is quickly followed by a second one, leaving the vortex core at the lower orbit $s_\text {min}$ and in the auto-oscillating regime. Since this double-reversals process is both periodical and sustained the vortex core is confined between these two orbits. This third regime is referred as the confinement regime and is only triggered for a vortex with a negative chirality.

## Methods

The vortex dynamics is studied by the means of micromagnetic simulations (MMS)^23^ using MuMax3. The free layer has a radius *R* of 500 nm, a thickness *t* of 9 nm and is discretized into cells of 2.5 × 2.5 × 4.5 nm^3^. The polarizer is on top of the free layer and its magnetization is fixed along $+e_z$. A magnetization saturation $M_s$ of 800 emu/cm^3^ and an exchange stiffness^24^$A_\text {ex}$ of 1.07 × 10^-6^ erg/cm corresponding to permalloy are used. The Gilbert damping constant $a_\text {G}$ is set to 0.01 and the spin-current polarization is $p_J =$ 0.2. By convention, a positive current flow along $+e_z$. The initial vortex polarity is set to $P=+$1. The temperature is set to $T =$ 0 K and no external magnetic field is applied. An input current density $J_\text {dc}$ between 0 and −10 MA/cm^2^ is applied to the STVO and the Ampère-Oersted field generated by the input current is added as an external magnetic field. For these simulations parameters, the STVO frequency is between 60 MHz and 120 MHz depending on the vortex chirality $C$ and the input current density $J_\text {dc}$. The evolution of the reduced vortex core position $s(t)$ with $s=||\textbf{X}||/R$ and $\textbf{X}$ the vortex core position, in the auto-oscillating regime extracted from MMS is fitted with the following equation^25^:

1 $$\begin{aligned} s(t) = \dfrac{s_\text {i}}{\sqrt{\left( 1 + \dfrac{s_\text {i} ^2}{(\alpha /\beta)}\right) e^{-2\alpha t} - \dfrac{s_\text {i} ^2}{(\alpha /\beta )}}} \end{aligned}$$

with $s_\text {i}$ the initial position of the vortex core and two parameters $\alpha$ and $\beta$ that depend on $J_\text {dc}$. To predict $s(t)$ for a given $J_\text {dc}$, the parameters $\alpha (J_\text {dc})$ and $\beta (J_\text {dc})$ are fitted with a linear fit: $\alpha (J_\text {dc}) = a_J J_\text {dc} + a$ and $\beta (J_\text {dc}) = b_J J_\text {dc} + b$ when the vortex core is in the auto-oscillating regime, see results in Table 1. In addition, the stable orbit $s_\infty$ is defined^25^ by:

2 $$\begin{aligned} s_\infty (J_\text {dc}) =&\sqrt{-\dfrac{\alpha (J_\text {dc})}{\beta (J_\text {dc})}} \end{aligned}$$

**Table 1:** Dynamical parameters of the considered model (see Eq. (1)) for the $C^-$ and $C^+$ chiralities.

Constant	$C^-$	$C^+$	Unit
$a_J$	−6.26	−12.49	Hz cm^2^ A^−1^
$b_J$	5.42	8.81	Hz cm^2^ A^−1^
$a$	−12.75	−13.47	MHz
$b$	−4.74	3.00	MHz

To predict the evolution of $s(t)$ in the confinement regime the parameters $s_\text {min}$ and $s_\text {max}$ are needed as well as four hypotheses: (1) the vortex polarity is reversed when $s \ge s_\text {max}$; (2) it is always followed by a second polarity reversal; (3) the double reversal process is instantaneous and 4) after the second reversal $s = s_\text {min}$. Both $s_\text {min}$ and $s_\text {max}$ can be fitted with a second order polynomial function. The combination of Eq. (1), the four hypotheses and the expression of the parameters $\alpha , \beta , s_\text {min}$ and $s_\text {max}$ depending on $J_\text {dc}$ allow predicting $s(t)$ for any current density as in Fig. 1. It gives the following recursive equation with $\Delta t$ the time step, $s_n$ and $s_{n-1}$ the current and previous reduced vortex core position respectively:

3 $$\begin{aligned} s_n = {\left\{ \begin{array}{ll} \dfrac{s_{n-1}}{\sqrt{\left( 1 + \dfrac{s_{n-1}^2}{(\alpha /\beta)}\right) e^{-2\alpha \Delta t} - \dfrac{s_{n-1}^2}{(\alpha /\beta )}}}, & \text {if } s_{n-1} <s_\text {max} \\ s_\text {min}, & \text {if } s_{n-1} \ge s_\text {max} \end{array}\right. } \end{aligned}$$

## Results and discussion

The two configurations where the vortex chirality $C$ is either positive or negative are studied. For $|J_\text {c1} | \le |J_\text {dc} | \le |J_\text {c2} |$, with $J_\text {c1}$ and $J_\text {c2}$ the first and second critical currents respectively, the vortex is in the steady-state regime. The evolution of the stable orbit $s_\infty$ with $J_\text {dc}$, depending on the vortex chirality, is predicted thanks to Eq. (2). The first critical current density $J_\text {c1}$ is defined by $\alpha (J_\text {c1}) =$ 0 leading to $J_\text {c1} = -a/a_\text {J}$. It gives $J_{\text {c1},C^+} =$ $-$1.08 MA/cm^2^ and $J_{\text {c1},C^-} =$ $-$2.04 MA/cm^2^ for a vortex with a positive and negative chirality respectively.

**Fig. 2:**
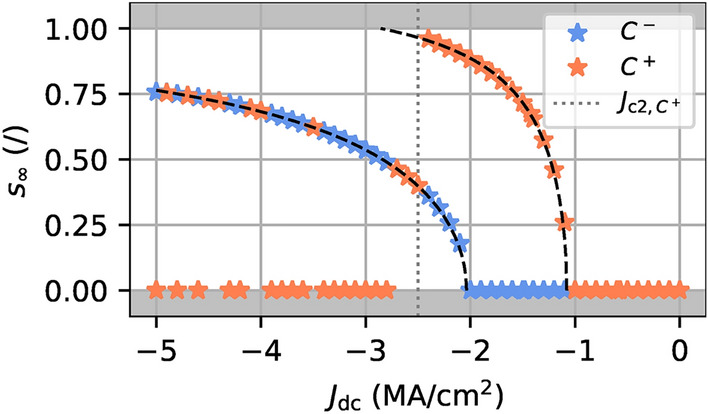
Evolution of the stable orbit $$s_\infty$$ with $$J_\text {dc}$$ for a negative (blue stars) or positive (orange stars) initial chirality. The second critical current $$J_{\text {c2},C^+}$$ is indicated by a dashed gray line. For an initial positive chirality, the vortex core reaches the magnetic dot limit for $$|J_\text {dc} | \ge |J_{\text {c2},C^+}|$$ with $$J_{\text {c2},C^+} \simeq$$$$-$$2.5 MA/cm^2^, its chirality is then reversed to $$C=-$$1 and the vortex core polarity is either unchanged, leading to a steady-state regime similar to the one of a vortex with an initial negative chirality, or reversed, resulting in a resonant regime. The black dashed lines are the prediction of $$s_\infty (J_\text {dc})$$ from Eq. (2).

The confinement regime is exclusively observed in vortices with negative chirality. For a positive initial chirality $C^+$ and $|J_\text {dc}| \ge |J_{\text {c2},C^+}|$, the vortex chirality is reversed to $C^-$ after the vortex core has been expelled from the nanodot, to align itself parallel to the Ampère-Oersted field generated by the current (see Fig. 2). The dynamics of the resulting vortex is therefore the same as if its initial chirality had been negative. Thus, it means that some orange stars are superimposed on blue stars because they share the same final orbit $s_\infty$, as explained in ref.^2^. However, it sometimes happens that in addition to a reversal of chirality, the polarity of the new vortex is also reversed. The $J_\text {dc}P p_z<$ 0 condition is therefore no longer met and the vortex falls back into a resonant regime. The core of the vortex then relaxes towards the centre of the nanodot to reach $s_\infty =$ 0.

**Fig. 3:**
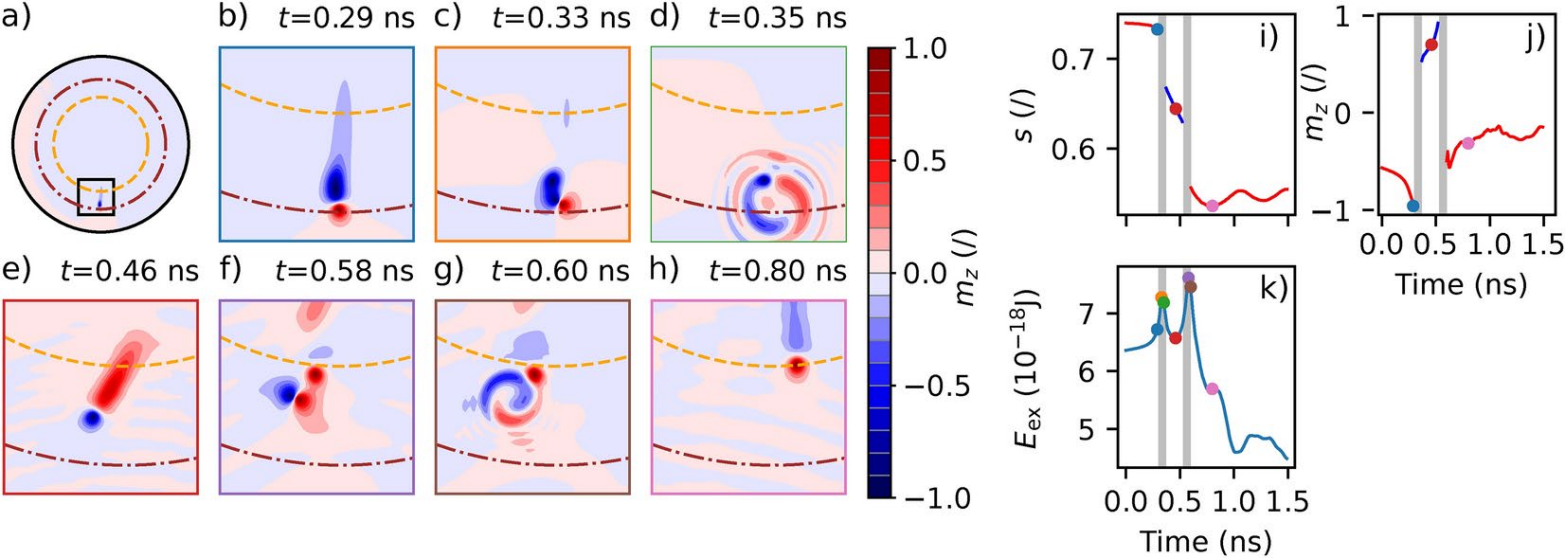
(**a**–**h**) Evolution of OOP component of the vortex magnetization with time. (**a**) View of the entire magnetic dot with the orbits $s_\text {min}$ and $s_\text {max}$ represented by orange dashed line and brown dashed-doted line respectively. (**b**–**h**) zoom of the magnetization delimited by the black box in (**a**). (**i**) Evolution of *s* as a function of time. The dot colours correspond to the border of the corresponding zoom from (**b**–**h**). Evolution of (**j**) the dip magnitude $m_z$ and (**k**) the exchange energy $E_\text {ex}$ as a function of time.

**Fig. 4:**
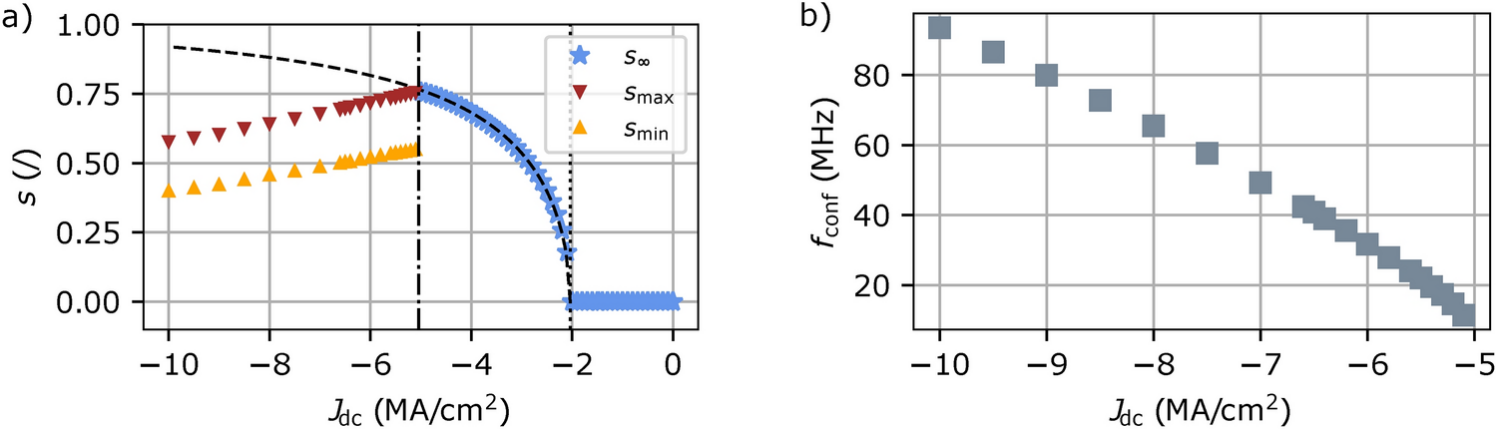
(**a**) Evolution of $s_\infty$, $s_\text {min}$ and $s_\text {max}$ with $J_\text {dc}$. The dashed line is the prediction of $s_\infty$ using Eq. (2). The dotted and dashed-dotted lines are $J_\text {c1}$ and $J_\text {conf}$ respectively. (**b**) Evolution of $f_\text {conf}$ with $J_\text {dc}$.

For a vortex with a negative chirality, when $$|J_\text {dc} | \ge |J_\text {conf} |$$ with $$J_\text {conf}$$$$\simeq {-}$$5.1 MA/cm^2^, the vortex core reaches its critical velocity at $$s = s_\text {max}$$ leading to its polarity reversal through the well-known process of creation and annihilation of a vortex-antivortex pair^17,26^. Indeed, the vortex core reaches a velocity of 285 ms^−1^ at $$s_\text {max}$$, which is coherent with the theoritical critical velocity^27,28^$$v_\text {cr} = \eta \gamma \sqrt{A_\text {ex}}=$$ 302 ± 32 ms^−1^ with $$\eta =$$ 1.66 ± 0.18 and $$\gamma =$$ 2π × 2.8 MHz/Oe, the gyromagnetic ratio. As the velocity of the vortex core increases, it undergoes a deformation with the development of a dip with an opposite magnetization (see Fig. 3). At the critical velocity^27^, the dip amplitude is the same as the amplitude of the vortex core and the vortex polarity is reversed, as shown in Fig. 3, through the creation and annihilation of a vortex-antivortex pair. The excess of exchange energy is then dissipated through spin waves generation^29^. As the polarity is then negative, the vortex core is in the resonant regime and relaxes towards its equilibrium position. It experiences a second polarity reversal as the new dip reaches the same amplitude as the vortex core and spin waves are once again generated. At the end of this second reversal, the vortex core is at $$s_\text {min}$$ and in the auto-oscillating regime again. This double reversal happens periodically, leading to a sustained confinement regime described by three parameters: $$s_\text {min}$$ and $$s_\text {max}$$ that define the lower and upper limits of the vortex core position, as well as the frequency $$f_\text {conf}$$ at which the double polarity reversals occur (see Fig. 4).

The orbits $$s_\text {min}$$ and $$s_\text {max}$$ decrease as the amplitude of $$J_\text {dc}$$ increases as shown in Fig. 4a. Indeed, the dip reaches the same amplitude as the vortex core for a lower orbit when $$J_\text {dc}$$ increases. The time between two double-reversal is extracted and the corresponding confinement frequency $$f_\text {conf}$$ is plotted in Fig. 4b. The frequency increases with $$J_\text {dc}$$, thus the double reversal frequency can be controlled by the dc input current with confinement frequencies starting at 11.5 MHz for $$J_\text {dc}$$$$=-$$5.1 MA/cm^2^ to 93.5 MHz for $$J_\text {dc}$$$$=-$$10.0 MA/cm^2^. Since spin waves are generated at each reversal, the frequency of spin wave generation is also controlled by $$J_\text {dc}$$.

## Conclusion

The dynamics of the vortex core is analytically described in both auto-oscillating and confinement regimes after fitting a few micromagnetic simulations. The emergence of the confinement regime depends on the vortex chirality and $J_\text {dc}$. Therefore, by adjusting the latter, the parameters of the confinement regime, namely $s_\text {min}$, $s_\text {max}$ and $f_\text {conf}$, can be controlled, offering the potential to fine-tune the properties of the confinement regime as well as the generation of spin waves. Finally, the confinement regime exhibits nonlinear and periodic dynamics, making it potentially valuable for neuromorphic computing applications^30^ as the vortex core remains in a transient regime and never reaches a stable orbit. It could be applied to leaky-integrate and fire neuron where the integration and leaking properties are controlled with the input current density and the firing correspond to the vortex core polarity reversal.

## Data Availability

The datasets generated during and/or analysed during the current study are available from the corresponding author on reasonable request.
